# Protecting the Public's Health in Pandemics: Reflections on Policy Deliberation and the Role of Civil Society in Democracy

**DOI:** 10.3389/fpubh.2021.678210

**Published:** 2021-07-06

**Authors:** Mary Beth Quaranta Morrissey, Jorge L. Rivera-Agosto

**Affiliations:** ^1^Global Health Care Innovation Management Center, Fordham University Gabelli School of Business, New York, NY, United States; ^2^Yeshiva University Wurzweiler School of Social Work, New York, NY, United States; ^3^Division of Medical Ethics, Department of Medicine, Northwell Health, New York, NY, United States

**Keywords:** pandemics, aging, public health, law, policy, ethics, government

## Abstract

The COVID-19 pandemic (“the pandemic”) has magnified the critical importance of public policy deliberation in public health emergency circumstances when normal health care operations are disrupted, and crisis conditions prevail. Adopting the lens of syndemic theory, the disproportionate impact of the pandemic on vulnerable older adults suggests that the pandemic has heightened pre-existing precarities and racial inequities across diverse older adult populations, underlining the urgency of needed policy reforms. While the pandemic has called attention to systemic failures in U.S. public health emergency planning at both federal and state levels of government, the important role of civil society in influencing policy decision making and advocating for legal and ethics reforms and social change in a democracy calls for more open dialogue in aging, public health and legal communities and constituencies. To foster this dialogue, one public health lawyer, who is also a bioethicist and gerontological social work researcher and served as chair of the New York State Bar Association Health Law Section COVID Task Force in 2020 (“Task Force”), shares her first-person perspectives on the process of leading the development of a statewide bar's recommendations for policy reforms, including the challenges and conflicts encountered. A hospital-based attorney and clinical bioethicist brings a clinical ethics perspective to the discussions. This first-person contribution discusses the power of constituencies to influence policy deliberation in a democracy, and the implications of the Task Force recommendations for future aging and public health policy, particularly in view of the high suffering burdens and trauma older persons and older people of color have borne during the pandemic.

## Introduction

The unprecedented magnitude and impact of the COVID-19 pandemic across diverse communities in the United States have foregrounded the critical role of civil society in processes of policy deliberation shaping aging and public health policy, especially in public health emergency circumstances when normal health care operations have been disrupted and crisis conditions prevail. Such crisis conditions of scarce resources that prevailed in the United States during the periods of the pandemic and posed the most serious threat to the public health, and before vaccines became widely available, heightened risk for vulnerable populations. The absence of clear uniform crisis standards of care to guide medical care decisions at the bedside created significant challenges for provider systems and physicians in struggling to meet the medical emergency needs of all those affected by COVID illness, especially vulnerable older persons and older people of color who experience barriers to care and, in some cases, discrimination in allocation of scarce resources at both system and bedside levels of care. It is also clear that in some cases, decisions have been based upon forms of systemic discrimination barred by law (1).

Adopting the lens of syndemic theory, the disproportionate impact of the pandemic on vulnerable older adults suggests that the pandemic has heightened pre-existing precarities and racial inequities in older adult populations, underlining the urgency of needed policy reforms. Syndemic theorizing sheds light on the biopsychosocial forces of the pandemic that have exposed such pre-existing inequities, including forms of systemic racism and ageism (2).

Against this backdrop of trauma, suffering and loss, as well as moral distress on the part of many health care workers, the pandemic has called attention to blistering systemic failures in public health emergency planning at both the federal and state levels of government. While there has been a major focus on the federal government and its failures (3), the important role of state government in regulating public health in the contexts of the threat to the public's health posed by COVID illness, including the balance of power between the executive and legislative branches, has not been well-understood. Fostering open and participatory dialogue about opportunities to advocate for policy reforms through the power and influence of diverse constituencies and advocates is a pillar of democracy and democratic processes of debate and deliberation.

### Reflections: The “Marketplace of Ideas” in Democracy

In looking back upon the experience of the last year, it's also helpful to reflect upon debates about the values that drive public health policymaking. These debates echo Thomas Jefferson' metaphor of the “marketplace of ideas” (4) that would create a space where Jefferson envisioned that reason would counter the “error of opinion,” a notion of truth-seeking advanced by Justice Oliver W. Holmes in arguing for freedom of speech protections (4, 5). In these contexts, it is evident that the public's acceptance of an organized public health response to the pandemic has been tempered by deeply entrenched attitudes toward and opinions about the role of state government and its legal authority to impose restrictions on the public, as well as skepticism about the very nature of public health itself and public health goals in protecting populations and communities. The task of balancing the competing goals of protecting the public's health and safeguarding civil liberties has been a source of constant and polarizing conflict during the pandemic, playing out in open challenges to states' imposition of requirements such as mask wearing, isolation, and quarantine. The authority of the state to protect the public in the contexts of a pandemic that poses a serious threat to the population rests upon a well-established body of constitutional law upholding the state's exercise of its police powers in regulating public health (6). Notwithstanding U.S. Supreme Court jurisprudence, however, historical tensions in the United States between the competing values of individual liberty, the market and small government on the one hand, and communitarian values that give primacy to the collective good of the society and social welfare on the other, have been front and center in debates about the role of government (3). Yet these debates are essential to a democracy and provide a ripe opportunity for all citizens and advocates to be heard and influence policy deliberation.

To address a gap in the public health literature vis-à-vis policy deliberations concerning public health law and regulation in pandemics and implications for aging and health policy making, two public health attorneys share their first-person perspectives on the New York pandemic narrative. As chair of the New York State Bar Association Health Law Section COVID Task Force in 2020, Morrissey charts her experience leading the development of policy recommendations adopted by the statewide bar, including the challenges and conflicts encountered, as well as policy successes, and follow-up conversations with state legislators. Rivera-Agosto speaks to challenges in the hospital systems from the perspective of a clinical bioethicist, providing rich context from the ground. Informed by these first-person perspectives, this contribution discusses the implications of the Task Force recommendations for future aging and public health policy, particularly in view of the high suffering burdens and trauma older persons and older people of color have borne during the pandemic. The disproportionate impact of the pandemic on older adults suggests that the pandemic has heightened both pre-existing precarities of older adults and inequities in allocation of resources to older adults across all settings, including nursing homes. Finally, the promise of palliative care is highlighted as a philosophy of care, an integrated medical, social and spiritual care intervention, and a critical component of effective public health strategy in mitigating suffering and trauma and fostering resilience in pandemics.

## New York's Pandemic Experience

The New York State Bar Association (NYSBA) Health Law Section COVID Task Force (“Task Force”) was appointed in early March 2020. The charge to the Task Force was to examine the key legal issues presented by the pandemic in the State of New York and under applicable New York law. The Task Force identified several key areas for study ranging from the role of state government in regulating the public health in emergencies to issues of structural racism and inequity across older adult populations.

Given the exigent circumstances in early March 2020, the Task Force worked feverishly to produce the first draft of its report by May 2020 and a final report by November 2020. The process of building consensus within both the Task Force itself and the wider state bar presented significant leadership challenges given the diversity of perspectives and interests and professional experience across the bar. Many of the challenges in building consensus related to proposed recommendations for major public health legal reforms in New York and calls for limits on the breadth of such proposed reforms. Concerns were raised about limiting executive powers in a public health emergency, as well as burdening civil liberties.

Dialogues were also held with key leaders in communities of color in the contexts of a decision structure proposed for guiding public health authorities on questions of whether vaccine mandates would be necessary in communities. The principal goals of such dialogues were to address issues of distrust in communities of color based upon contemporary racism and the U.S. history of exploitation of people of color in research studies, and develop strategies to encourage public acceptance and uptake of vaccines that had been approved by regulatory authorities.

Given the broad consensus that was achieved during months of intensive work, the Task Force Report (“Report”) as a whole and the final set of Resolutions and recommendations adopted by the statewide bar demonstrate an unparalleled breadth and depth of inquiry and deliberation about public health law and policy in the real-time contexts of the pandemic. The key provisions of the Resolutions (7) are outlined below:

- Enact a state emergency health powers act addressing gaps in existing laws in New York, drawing upon the Model State Emergency Health Powers Act (MSEHPA), developed by the Center for Law and Public Health at Georgetown and Johns Hopkins Universities (8, 9), and other sources as appropriate;
- Adopt crisis standards of care addressing gaps in existing laws in New York, drawing upon the Crisis Standards of Care, developed by the Institute of Medicine (10); The Arc, Bazelon Center for Mental Health Law, Center for Public Representation, Disability Rights Education and Defense Fund, and Autistic Self Advocacy Network Evaluation Framework for Crisis Standard of Care Plans (11) (“Evaluation Framework”); and other sources as appropriate;
- Provide comprehensive workforce education and training in the implementation of the above state emergency health powers act and crisis standards, including proper use and disposal of PPE and other equipment;
- Appoint and maintain a core team of emergency preparedness experts to review evidentiary sources and draft legislation to strengthen emergency preparedness planning;
- Adopt resource allocation guidelines addressing gaps in existing laws in New York, drawing upon the New York State Task Force on Life and the Law 2015 Report, Ventilator Allocation Guidelines (12), the Evaluation Framework, and other sources as appropriate; and
- Issue emergency regulations mandating all providers and practitioners follow the ethics guidelines, and ensure:
  ° the needs of vulnerable populations, including persons and communities of color, older adults and nursing home residents, persons with disabilities, persons who are incarcerated, and immigrants, are met in a nondiscriminatory manner in the implementation of emergency regulations and guidelines;
  ° provision of palliative care to all persons as an ethical minimum to mitigate suffering among those who are in institutional, facility, residential, or home care settings during the COVID-19 crisis;
  ° provision of education and training to physicians, health care practitioners, and institutional triage and ethics committees; and
  ° provision of generalist-level palliative care education and training for all health care workers and health-related service workers in all settings who are providing supportive care.

The following selected issue areas addressed in the November 2020 Report and Resolutions, and by a second NYSBA Nursing Home and Long-Term Care Task Force,^1^ are discussed more fully below: (i) the proposed public health legal reforms and their scope; (ii) ethical issues in the allocation of scarce resources; (iii) long-term care systems and impact of the pandemic upon nursing home residents; and (iv) vulnerable populations and equitable access to palliative care, virus testing and vaccination.

### Public Health Legal Reforms

The Report recommends major public health legal reforms in New York, including enactment of a state emergency health powers act and adoption of crisis standards of care (13). Drawing on the MSEHPA (8, 9), the Report recommends that clear statutory authority in emergencies is critically important. Such clear statutory authority would perhaps have avoided action taken by the legislature investing broad emergency powers in the executive branch of state government, and the reliance in New York State on executive orders and guidance during the pandemic (14). Similarly, adoption of crisis standards of care, drawing on the Institute of Medicine Model Crisis Standards of Care (10), would have provided clear guidance on decision making during the pandemic following widely accepted ethical principles. The Resolutions adopted (7) clarify that the recommendations made do not call for wholesale enactment or adoption of model acts and standards, but rather crafting of provisions that fit the needs of New York State and swift action to put these measures in place in the present pandemic, as well as for the purposes of preparing for future public health emergencies.

### Ethical Issues in Allocation of Scarce Resources

Extensive discussions not only within the bar, but with physicians and bioethicists in major hospital systems both upstate and downstate, were had regarding scarce resources during the pandemic, including allocation of ICU beds, PPE and staffing (15). Debate centered around not only who would get what resources when there was not an adequate supply to meet the needs of all patients, but who would decide how scarce resources would be allocated. Would such decisions be made at the bedside by practitioners, or would there be clearly articulated guidelines that practitioners on the ground could follow? Despite a strong consensus that it was the role of the state to issue triage guidelines and urging by bioethicists for the state to take action, no such guidelines were issued in New York, leaving many practitioners in the position of making decisions on their own. For example, great controversy surrounded whether physicians could make determinations not to resuscitate based upon their own clinical judgments even if such determinations ran counter to the express wishes of the family or surrogate (16). For example, the Report identifies and recommends adoption of certain procedural protections by health providers in the case of futility DNR Orders:

More specifically, we recommend that any disaster or emergency crisis-related futility DNR should still be subject to certain procedural protections, for example, (i) futility must be defined narrowly, in terms of effectiveness of restarting the heart, as it is in PHL 2991; (ii) there must be a concurring determination of medical futility by a second practitioner, selected by the facility; (iii) the attending practitioner must notify the patient or, if the patient lacks capacity, the agent/surrogate of the order and the basis for it; (iv) such determinations must be documented in the medical record; and (v) if the order is issued without patient/agent or surrogate consent, there should be a post-issuance medical peer review of the medical support for the futility finding (13).

Questions about discrimination in allocation of scarce resources to older persons and persons with disabilities remained an ongoing concern throughout the pandemic, including explicit rationing decisions, as well as implicit forms of rationing, for example, in failures to equip nursing homes with adequate PPE and staffing during the pandemic (16).

### Long-Term Care Systems and Disproportionate Impact Upon Nursing Home Residents

It is now well-known that the pandemic imposed unforeseeable burdens on providers who were ill-equipped to meet the needs of patients. The long-term care systems in New York, and in other states, including psychiatric hospitals and other congregate care settings, were hard hit, but perhaps no population was more detrimentally impacted by the pandemic than those older persons residing in New York's nursing homes, especially older people of color. Research study findings suggest nursing homes with higher proportions of non-White residents were more likely to experience COVID-19 cases and/or deaths (17–19). Other facility characteristics have been positively associated with the high number of deaths in nursing homes, including ownership and low levels of staffing (20). However, the results of a targeted analysis of New York data made recently available through a FOIL request suggest that the evidence may be mixed and less than persuasive on the question as to whether low staffing or for-profit ownership contributed to New York's nursing home mortality (21).

From a more global policy perspective, while pre-pandemic and historical policy failures at the federal level of government left many nursing homes and nursing home residents more vulnerable to the pandemic (22), macro-level policy decisions at the state level of government in New York both before and during the pandemic also contributed significantly to the number of deaths, including: (i) historical underinvestment in New York's public health infrastructures and systems (23); (ii) historical underfunding of nursing homes and levels of reimbursement; (iii) failure to allocate adequate PPE to nursing homes during the pandemic (24); and (iv) issuance of Executive Orders and Guidance, including the March 25, 2020 guidance directing that COVID positive nursing home residents be transferred from hospitals to nursing homes (25, 26), that detrimentally affected under-resourced nursing homes, and most importantly, the nursing homes residents themselves who suffered the trauma of isolation.

### Vulnerable Older Adult Populations and Equitable Access to Palliative Care, Virus Testing, and Vaccination

The 2020 NYSBA COVID Report and Resolutions speak throughout to pre-existing inequities in social and economic determinants of health that have heightened suffering of older persons during the pandemic. In response to such historical inequities, recommendations have been made to ensure equitable access to care for all older adults and vulnerable populations, especially older communities of color who have been disproportionately impacted by the pandemic. Such recommendations call for older adults' and nursing homes residents' priority access to virus testing and vaccination. Older persons residing in correctional facilities and older immigrants are included in the scope of the recommendations. Importantly, the recommendations also call for strengthening palliative health and social services and supports as an ethical minimum of care during pandemics, consistent with the provisions of the Institute of Medicine Model Crisis Standards of Care (10). Reframing palliative care as essential integrated medical and social care (27) during a pandemic is critically important in mitigating pain and suffering, and in responding to experience of massive losses, trauma, and bereavement.

## Discussion

First-person perspectives of leadership as demonstrated by a statewide bar association in New York yield insights about the important role of civil society (28) in fostering policy deliberations through processes of debate and consensus-building, culminating in final recommendations for certain public health legal and ethics reforms in New York. These recommendations emerged from a broad consensus that the pandemic has exposed and heightened structural racism in the United States, described by some scholars as the racism pandemic (2), and pre-existing inequities and intersectional health disparities by race, ethnicity and age (22, 29). Syndemic theory (2) advances understanding of interaction and concentration of disease and macro-level sociopolitical and economic forces, including systemic racism and ageism, that have contributed significantly to suffering and mortality during the pandemic, and may guide the formulation of public health policy. In addition to the specific recommendations made in the New York State Bar Association Report and Resolutions (7, 13), reflections by two public health law attorneys and bioethicists on the ground in New York during the pandemic provide first-person perspectives on the challenges that were faced in the course of intensive work over many months to build support across diverse constituencies for a plan of action to address the urgent needs of communities. Consistent with recommendations made by other leaders, in their global advocacy for a robust public health response to the pandemic in New York, public health lawyers and policy advocates prioritized the values of equality, equity, adequacy and justice, calling for dialogues with key leaders in communities of color, eliminating disparities, strengthening public health infrastructures, and ensuring equitable access to palliative care as an ethical minimum for all persons. Recent conversations with state legislators show some promise that at least certain recommendations made by the statewide bar may be taken seriously. The work done in New York by a professional association in dialogue with health care professionals and civic leaders may serve as a model for other states in public health planning and research for the purposes of developing policy reforms that address the present humanitarian crisis as well as future threats to the public's health.

## Data Availability Statement

The original contributions presented in the study are included in the article/supplementary material, further inquiries can be directed to the corresponding author/s.

## Author Contributions

MBQM wrote the manuscript. JLR-A substantially contributed to the Ethics section. MBQM and JLR-A edited the manuscript. All authors contributed to the article and approved it for publication.

## Conflict of Interest

The authors declare that the research was conducted in the absence of any commercial or financial relationships that could be construed as a potential conflict of interest.

## Footnotes

^1^Mary Beth Morrissey was appointed to the New York State Bar Association Nursing Home and Long-Term Care Task Force in July 2020.

## References

[1] U.S. Department of Health and Human Services – Office for Civil Rights. OCR Reaches Early Case Resolution with Alabama After It Removes Discriminatory Ventilator Triaging Guidelines (2020). Available online at: https://www.hhs.gov/about/news/2020/04/08/ocr-reaches-early-case-resolution-alabama-after-it-removes-discriminatory-ventilator-triaging.html (accessed April 29, 2021). For Alabama, Utah, and Pennsylvania: U.S. Department of Health and Human Services – Office for Civil Rights. Civil Rights and COVID-19 (2021). Available online at: https://www.hhs.gov/civil-rights/for-providers/civil-rights-covid19/index.html (accessed April 29, 2021).

[2] Gravlee CC. Systemic racism, chronic health inequities, and COVID-19: a syndemic in the making? Am J Hum Biol. (2020) 32:e23482. 10.1002/ajhb.23482 32754945 PMC7441277

[3] Woolhandler S Himmelstein DU Ahmed S Bailey Z Bassett MT Bird M. Public policy and health in the Trump era. Lancet Commissions. (2021) 397:705–53. 10.1016/S0140-6736(20)32545-9 33581802

[4] Thomas Jefferson. The Papers of Thomas Jefferson (2006). p. 148–52. Available online at: https://jeffersonpapers.princeton.edu/volumes/volume-33 (accessed April 29, 2021).

[5] Abrams v. U.S., 250 U.S. 616, 630 (1919) (Holmes, J., dissenting).

[6] Jacobson v. Massachusetts, 197 U.S. 11 (1905).

[7] New York State Bar Association. New York State Bar Association COVID-19 Resolutions (2020). Available online at: https://nysba.org/app/uploads/ 2020/02/Final-NYSBA-COVID-19-Resolutions_11-7-20-with-single-link.pdf (accessed March 8, 2021).

[8] The Center for Law and the Public's Health at Georgetown and Johns Hopkins Universities. The Model State Emergency Health Powers Act (2001). Available online at: https://www.aapsonline.org/legis/msehpa.pdf (accessed March 8, 2021).

[9] Speissegger S Runyon C. The Model State Emergency Health Powers Act: A Checklist of Issues. Washington, D.C.: National Conference of State Legislatures (2002).

[10] Institute of Medicine. Crisis Standards of Care: A Systems Framework for Catastrophic Disaster Response: Volume 1: Introduction and CSC Framework. Washington, DC: The National Academies Press (2012). 24830057

[11] The Arc Bazelon Center for Mental Health Law Center for Public Representation Disability Rights Education and Defense Fund and Autistic Self Advocacy Network. Evaluation Framework for Crisis Standard of Care Plans (2020). Available online at: https://www.centerforpublicrep.org/wp-content/uploads/Updated-evaluation-framework.pdf (accessed March 8, 2021).

[12] New York State Task Force on Life and the Law. Ventilator Allocation Guidelines (2015). Available online at: https://www.health.ny.gov/regulations/task_force/reports_publications/docs/ventilator_guidelines.pdf (accessed March 8, 2021).

[13] New York State Bar Association. New York State Bar Association Health Law Section COVID-19 Report (2020). Available online at: https://nysba.org/app/uploads/2020/09/Health-Law-Section-COVID-19-Report-September-20-2020.pdf (accessed March 8, 2021).

[14] New York State Office of the Governor. Executive Orders (2021). Available online at: https://www.governor.ny.gov/executiveorders (accessed March 8, 2021). We bring particular attention to Executive Orders 202, 202.5, 202.10, 202.11, 202.18, 202.19, 202.23, 202.30, 202.32, 202.40, 202.44, 202.60, 202.73, 202.77, 202.88, and their respective renewals and modifications.

[15] Fins JJ. Is deliberative democracy possible during a pandemic? Submitted to the APA Journal of Theoretical and Philosophical Psychology. In: Paper Presented at the New York State Bar Association's “Clinical, Legal, Ethical Considerations in Deliberative Democracy in a Pandemic” Continuing Legal Education Program on April 23, 2021 (2021).

[16] Kisner J. What the Chaos in Hospitals Is Doing to Doctors (2021). Available online at: https://www.theatlantic.com/magazine/archive/2021/01/covid-ethics-committee/617261/ (accessed March 8, 2021).

[17] Chidambaram P Neuman T Garfield R. Racial and Ethnic Disparities in COVID-19 Cases and Deaths in Nursing Homes (2020). Available online at: https://www.kff.org/coronavirus-covid-19/issue-brief/racial-and-ethnic-disparities-in-covid-19-cases-and-deaths-in-nursing-homes/ (accessed May 6, 2021).

[18] Ochieng N Chidambaram P Garfield R Neuman T. Factors Associated With COVID-19 Cases and Deaths in Long-Term Care Facilities: Findings from a Literature Review (2021). Available online at: https://www.kff.org/coronavirus-covid-19/issue-brief/factors-associated-with-covid-19-cases-and-deaths-in-long-term-care-facilities-findings-from-a-literature-review/#:~:text=These%20nine%20studies%20found%20that,117%20and%20severity%20of%20outbreak (accessed March 8, 2021).

[19] Gorges R Konetzka RT. Factors associated with racial differences in deaths among nursing home residents with COVID-19 infection in the US. JAMA Network Open. (2021) 4:e2037431. 10.1001/jamanetworkopen.2020.37431 33566110 PMC7876590

[20] Abrams HR Loomer L Gandhi A Grabowski DC. Characteristics of U.S. nursing homes with COVID-19 cases. JAGS. (2020) 68:1653–6. 10.1111/jgs.16661 32484912 PMC7300642

[21] Empire Center. COVID Nursing Home Data (2021). Available online at: https://www.empirecenter.org/publications/covid-nursing-home-data/ (accessed May 6, 2021).

[22] Morrissey MBQ Brownell P Caprio T. Intersectionality of race, ethnicity, and culture in neglect, abuse, and violence against older persons: human rights, global health, and systems approaches in pandemics. In: Geffner R, White JW, Hamberger LK, Rosenbaum A, Vaughan-Eden V, Vieth VI, editors. Handbook of Interpersonal Violence and Abuse Across the Lifespan. Cham: Springer (2020).

[23] Hammond B. Bill Hammond Testifies on Public Health Spending in FY2022 Budget (2021). Available online at: https://www.empirecenter.org/publications/bill-hammond-testifies-on-public-health-spending-in-fy2022-budget/ (accessed March 8, 2021).

[24] SEIU. Issue Brief: Personal Protective Equipment (PPE) (2020). Available online at: https://www.1199seiu.org/education (accessed March 8, 2021).

[25] New York State Department of Health. Advisory: Hospital Discharges and Admissions to Nursing Homes (2020). Available online at: https://skillednursingnews.com/wp-content/uploads/sites/4/2020/03/DOH_COVID19 NHAdmissionsReadmissions 032520_1585166684475_0.pdf (accessed March 8, 2021).

[26] Hammond B Kingsbury I. Implications of COVID-19 Mortality Patterns for Nursing Home Regulation in New York (2021). Available online at: https://www.empirecenter.org/publications/implications-of-covid-19-mortality-patterns/ (accessed March 8, 2021).

[27] Morrissey MB Herr K Levine C. Public health imperative of the 21st Century: innovations in palliative care systems, services, and supports to improve health and well-being of older Americans. Gerontologist. (2015) 55:245–51. 10.1093/geront/gnu178 26035600 PMC6282687

[28] Jennings B Gusmano MK Kaebnick GE Neuhaus GP Solomon MZ. Recommendations for better civic learning: building and rebuilding democracy. Hastings Center Rep. (2021) 51:S64–75. 10.1002/hast.1232 33630335

[29] Samra R Hankivsky O. Adopting an intersectionality framework to address power and equity in medicine. Lancet. (2021) 397:857–9. 10.1016/S0140-6736(20)32513-7 33357466 PMC9752210

